# Aluminium co-localises with Biondi ring tangles in Parkinson’s disease and epilepsy

**DOI:** 10.1038/s41598-022-05627-8

**Published:** 2022-01-27

**Authors:** Matthew John Mold, Christopher Exley

**Affiliations:** The Birchall Centre, Lennard-Jones Laboratories, Keele University, Keele, Staffordshire, ST5 5BG UK

**Keywords:** Neuroscience, Biochemistry, Bioinorganic chemistry, Protein folding, Mechanisms of disease, Microscopy

## Abstract

Aluminium is known to accumulate in neuropathological hallmarks. However, such has only tentatively been suggested in Biondi ring tangles. Owing to their intracellular and filamentous structure rich in β-pleated sheets, Biondi ring tangles might attract the adventitious binding of aluminium in regions of the blood–cerebrospinal fluid barrier. The study’s objective was to establish whether aluminium co-localises with Biondi ring tangles in the brains of Parkinson’s disease donors versus a donor that went on to develop late-onset epilepsy. Herein, we have performed immunohistochemistry for phosphorylated tau, complemented with aluminium-specific fluorescence microscopy in the choroid plexus of Parkinson’s disease donors and in a donor that developed late-onset epilepsy. Aluminium co-localises with lipid-rich Biondi ring tangles in the choroid plexus. While Biondi ring tangles are not composed of phosphorylated tau, the latter is identified in nuclei of choroidal cells where aluminium and Biondi ring tangles are co-located. Although Biondi ring tangles are considered artefacts in imaging studies using positron emission tomography, their ability to bind aluminium and then release it upon their subsequent rupture and escape from choroidal cells may allow for a mechanism that may propagate for aluminium toxicity in vivo.

## Introduction

Parkinson’s disease (PD) is a progressive neurodegenerative disorder characterised neuropathologically by Lewy bodies of aggregated α-synuclein and the loss of dopaminergic neurons in the substantia nigra^1^. A complex interplay between genetic and environmental risk factors are implicated in the disease process of which mutations in leucine-rich repeat kinase 2 (LRRK2) and the E3 ubiquitin ligase, parkin, are those most commonly associated with familial PD^1,2^. While mutations in LRRK2 are associated with disrupted autophagy pathways, mutations in parkin and the phosphatase and tensin homolog-induced putative kinase 1 (PINK1) gene are responsible for impaired mitophagy^3^. Lysosomal and proteasomal pathways are crucial in maintaining normal cellular physiology, and their failure leads to the increased accumulation of reactive oxygen species (ROS) and neuronal death in PD^1,3^.

The amyloidosis disorders share an accumulation of misfolded proteins, of which aberrant processing of their soluble random coil and α-helical precursors leads to aggregated protein inclusions defining the disease status^4^. In PD, Lewy bodies form as inclusions in neuronal cells, predominantly resulting in vesicular aggregation through impaired cellular clearance mechanisms^5^. However, these pathological hallmarks are rarely found in isolation of which concomitant neuronal loss and pathological similarities to Alzheimer’s disease (AD) have been reported^1,4^. Furthermore, in both AD and PD, extracellular senile plaques of amyloid-β (Aβ) and intracellular neurofibrillary tangles (NFTs) of phosphorylated tau have been identified, and in PD, their presence can synergistically exacerbate α-synuclein pathology^4^.

The hippocampus is an important target in epilepsy. Neuropathological examination of the brains of donors with PD, AD and temporal lobe epilepsy (TLE) has shown extensive phosphorylated tau deposition in NFTs in the hippocampus versus donors that died of non-neurological causes^6^. Reduced excitability of pyramidal neurons in the cornu ammonis area (CA) 1 of the hippocampus has been reported in transgenic mouse models of hyperphosphorylated tau^7^. Such modulations in neuroplasticity are relevant to epileptogenesis, of which hippocampal sclerosis marked by neuronal cell loss in the CA1 region is also observed in excised brain tissue from TLE donors^8^. Elevated Aβ_42_ comprising the principal pathogenic component of senile plaques in AD, is also elevated and restricted to the hippocampus of donors with drug-resistant TLE^4,6^.

Progressive α-synuclein pathology and atrophy of the hippocampus have prompted investigations in living PD patients to identify biomarkers of early disease onset and to discriminate mild cognitive impairment (MCI) associated with related neurodegenerative conditions^9,10^.

To this end, the use of the fluorescent radiolabel, ^18^F-flortaucipir (^18^F-AV-1451), has allowed for the spatial distribution of NFTs of phosphorylated tau to be assessed via positron emission tomography (PET)^11^. Early studies using the benzimidazole-based radiotracer identified the binding mode to paired helical filaments (PHFs) of pathological tau tangles. Such was confirmed by positive immunolabelling against the monoclonal antibody, AT8, targeting tau phosphorylated on residues Ser202 and Thr205^12^. Furthermore, ^18^F-flortaucipir demonstrates high specificity to NFTs of phosphorylated tau, readily distinguished from fibrillar Aβ in senile plaques that deposit in the same cortical brain regions^12–14^. For the latter, ^18^F-florbetapir has allowed for immunotherapeutic and disease-modifying agents, including the monoclonal antibody, aducanumab, to be successfully monitored in clinical trials that reduce Aβ plaque load^15^.

The use of PET to monitor neurodegenerative disease progression has generally been restricted to sporadic and early-onset familial forms of AD^12–16^. To date, no suitable fluorescent radioligand for PET has been developed to allow for clinical assessments of α-synuclein propagation as Lewy body pathology in PD^17^. However, recent advances in high throughput in silico screening of an extensive database of potential radioligands have identified potential candidates for future use in clinical trials^18^. Furthermore, the use of ^18^F-flortaucipir PET has allowed for donors with progressive supranuclear palsy (PSP) to be delineated from those with PD^11^. As a divergent parkinsonian disorder, PSP is marked by the loss of cognitive and motor functions with a high tau burden in the limbic system of affected patients^11^. Despite the clear advantages offered by PET, both clinical assessments of neurodegenerative disease progression in living patients and brain tissues performed at autopsy are susceptible to “off-target” tracer binding^11–14^.

The hippocampus and, in particular, the choroid plexus is known to produce high backgrounds to the ^18^F-flortaucipir radiotracer^19,20^. Epithelial cells that line the choroid plexus produce cerebrospinal fluid (CSF), and the tight junctions formed between adjacent cells form the blood–CSF barrier (BCSFB). The choroid plexus is situated in the innermost pia mater layer of the meninges and sits close to the spinal cord and cerebral cortex. This highly organised tissue structure is formed by specialised ependymal glial-like cells that line all brain ventricles except the frontal/occipital horn of the lateral ventricle and cerebral aqueduct^21^. These cuboidal epithelial cells possess cilia or hair-like projections that both increase the surface area of the choroid plexus and enhance reabsorption of CSF. In the hippocampus, the choroid plexus interdigitates between the tail of the caudate nucleus and the fimbria and fimbriodentate fissure and is located in the temporal horn of the lateral ventricle^21^.

Lipid-rich lipofuscin deposits accumulate intracellularly in choroid plexus epithelial cells upon ageing, notably in donors with neurodegenerative disease, including AD and PD. Over time, these cells become flattened, and thickening of connective tissues in the stromal and basement membrane occurs^19^. Subsequent histological and scanning electron microscopy (SEM) analyses of excised tissues have revealed the presence of lipid-rich filamentous tangles, known as Biondi ring tangles^19,20,22,23^. Those tangles formed also produce fluorescence in the presence of the benzothiazole-based stain, thioflavin S (ThS), that is known to bind to β-pleated sheets of amyloid fibrils^20^.

Paradoxically, given the high number of transporters for Aβ expressed in choroidal epithelial cells and ventricular ependymal cells, the accumulation of intracellular Aβ in these cells would seem surprising^24^. Immunohistochemistry against Aβ has been shown to produce no reactivity to Biondi ring tangles. In addition, the on-target binding of ^18^F-flortaucipir and AT8-reactivity of Biondi tangles supports the consensus for their principal constituents to be that of phosphorylated tau in the form of NFTs^25^. While Biondi tangles are frequently noted in the brains of donors with AD, we have previously made such observations in donors with PD and in a donor that developed late-onset epilepsy^26,27^. In PD, Biondi ring tangles produced positive ThS fluorescence and were tentatively co-localised with aluminium in epithelial cells of the choroid plexus^26^. As a non-essential yet ubiquitous metal in the environment, the biologically reactive form of aluminium, Al^3+^_(aq)_, is neurotoxic and is universally associated with amyloid-β in senile plaque cores and to a lesser degree with phosphorylated tau in NFTs in AD brain tissue^28,29^. Aluminium was found to accumulate in epithelial cells lining the choroid plexus of a donor with severe cerebral amyloid angiopathy (CAA) after exposure to high levels of aluminium in their potable water supply^30,31^. Acute aluminium toxicity experienced by a second 60-year-old male donor who developed late-onset epilepsy revisited in this study, demonstrated numerous Biondi ring tangles in the choroid plexus and surrounding ventricular spaces in the hippocampus with minimal evidence for an association with aluminium^27^. Previously, we observed notably high aluminium levels in this donor's hippocampus as supported by aluminium-specific fluorescence microscopy^27^. Thorough neuropathological examinations of donors exposed to high levels of aluminium in their potable water supply in the 1988 Camelford incident have been made elsewhere^31,32^.

Herein, we have performed aluminium-specific fluorescence microscopy in the choroid plexus of PD donors and in a donor that went on to develop epilepsy following an acute exposure to aluminium. Such was performed cases to address the tentative suggestion of aluminium binding to Biondi ring tangles in these disease states^26^. Lumogallion is a sensitive and selective fluorescent molecular probe for the detection of aluminium in vivo. We have extensively optimised lumogallion for the unequivocal detection of aluminium in human brain tissue. Lumogallion does not produce a fluorescent bond when coordinated to common interfering yet physiologically relevant concentrations of Fe(III)^33^. The same is true for Zn(II), Ca(II) and Mg(II)^33,34^. We have further supported these observations by impregnating surrogate brain tissue with a range of transition metal ions and processing them as real tissue^35^. Aluminium is observed as a characteristic orange fluorescence emission that is neither reproduced by other metals nor explained by autofluorescence^35^.

Increased brain aluminium content and its association with neuropathological hallmarks of disease have been reported in neurological conditions that exert inevitable neurotoxicity^36,37^. While Biondi ring tangles are frequently observed in choroid plexus epithelial and ventricular ependymal cells, their presence is often overlooked or ignored as “off-target” labelling of the radiotracer in PET analyses^19–23,25^. Therefore, we have additionally investigated Biondi ring tangles via immunohistochemistry, ThS and UV-induced protein autofluorescence to determine their potential co-localisation to aluminium in tissues of the choroid plexus.

## Materials and methods

### Human brain tissue

All experimental protocols and ethical approval was obtained along with brain tissues from anonymised donors diagnosed with Parkinson’s disease (PD) from the Parkinson’s UK Brain Bank at Imperial College, London, funded by Parkinson’s UK (NREC approval no. 18/WA/0238), as approved by Keele University, UK. Human brain tissue was received as 5 µm thick adjacent serial sections (five of each on electrostatically charged glass slides) prepared from formalin-fixed paraffin-embedded (FFPE) tissue blocks. PD brain tissue included two female donors aged 67 and 76-years-old, respectively. Brain tissue provided as FFPE blocks from a 60-year-old male who died following asphyxiation brought on by an epileptic fit was provided by University Hospitals Plymouth, NHS Trust, UK. Written informed consent was obtained from the patient’s next of kin, and all methods were carried out in accordance with relevant guidelines and regulations. Tissue blocks were provided to Keele University upon the request of the coroner to investigate the content and distribution of aluminium. In 1988, the deceased, as described by the coroner, was exposed to high levels of aluminium in his potable water supply as a victim of the Lowermoor Treatment Works pollution disaster in Camelford, Cornwall, UK. Full pathological details of this case are described elsewhere^27^.

### Microtomy

FFPE hippocampal brain tissue from a male donor with late-onset epilepsy mounted on Tissue-Tek embedding cassettes (VWR, Sakura Finetek, Harrisburg, PA, USA) was cooled for 10 min on wet ice to increase tissue hardness minimising imperfections upon sectioning. Adjacent and numbered serial sections were prepared at a thickness of 5 µm using a rotary Leica RM2025 microtome equipped with low profile stainless steel Surgipath DB80LX blades (Leica Microsystems Ltd, UK). Sections were floated onto ultrapure water (conductivity < 0.067 µS cm^−1^) in a water bath maintained at 40 °C, allowing for gentle tissue expansion. Sections were transferred onto SuperFrost Plus electrostatically charged adhesion glass slides (Thermo Fisher, UK) and were dried vertically at ambient temperature. Before use, sections were heated at 62 °C for 20 min to remove excess paraffin, increasing tissue adherence.

### Deparaffinisation and rehydration of brain tissue sections

Deparaffinisation of human brain tissue sections was achieved through immersion in Histo-Clear (National Diagnostics, USA) for 3 min, followed by 1 min in fresh Histo-Clear. The latter was performed to remove any remaining wax. Histo-Clear was removed by immersion in pure ethanol (Fisher Scientific, UK; HPLC grade used for all procedures) for 2 min. Sections were rehydrated through an ethanol gradient for 1 min in each solvent. Sections were washed twice with ultrapure water for 30 s to remove any remaining ethanol before staining procedures. Gentle agitation was performed at every stage to provide a thorough removal of any prior solvent.

### Lumogallion staining

Rehydrated tissue sections were outlined with a hydrophobic PAP pen before being transferred to humidity chambers for all subsequent staining procedures. Sections were incubated in 1 mM lumogallion (4-chloro-3-)2,4-dihydroxyphenylazo)-2-hydroxybenzene-1-sulphonic acid TCI Europe N. V., Belgium) in 50 mM PIPES, pH 7.4. The adjacent serial section was incubated in 50 mM PIPES, pH 7.4 only, to assess autofluorescence. All sections were incubated for 45 min at ambient temperature and away from light. Staining was performed using staggered time intervals to ensure all sections were stained for the same duration. Following staining, tissue sections were washed six times with fresh 50 mM PIPES buffer and rinsed for 30 s in ultrapure water. While moistened, brain tissue was mounted with Fluoromount (Sigma Aldrich, UK), an aqueous-based mounting medium.

### Thioflavin S staining

Lumogallion-stained tissue sections were fully immersed in ultrapure water with gentle agitation overnight to remove mounted coverslips. Thioflavin S (ThS) was prepared at ca 0.075% w/v in 50% v/v ethanol immediately before use. Lumogallion-stained sections were counter-stained with ThS for 8 min at ambient temperature, away from light. Sections were subsequently washed twice for 10 s in fresh solutions of 80% v/v ethanol and rinsed by gentle agitation for 30 s in ultrapure water. Coverslips were mounted with Fluoromount.

### Immunohistochemistry

All reagents for immunostaining were obtained from Abcam, UK unless otherwise stated. Immunohistochemistry (IHC) was performed against human phosphorylated tau (Ser202 and Thr205) using a rabbit monoclonal AT8 primary antibody (ab210703, 0.472 mg/mL). Sections for IHC were outlined with a hydrophobic PAP pen, and all labelling procedures were carried out in humidity chambers to prevent tissues from drying out. All washing procedures were performed using gentle agitation. Antigen retrieval was performed enzymatically using Proteinase K antigen retrieval solution (ab64220) via incubation for 7 min at ambient temperature. Sections were twice washed for 5 min in 1 × Tris-buffered saline (TBS), pH 7.4, followed by two washes in the same buffer containing 0.025% v/v Triton X-100 (Sigma Aldrich, UK), for 5 min. Blocking was performed using 10% normal goat serum with 1% w/v bovine serum albumin (BSA) in 1 × TBS buffer for 2 h at ambient temperature.

The AT8 primary antibody was diluted 1:50 in 1% w/v BSA in 1 × TBS buffer, and sections were incubated overnight at 4 °C to ensure complete tissue saturation and antibody binding. Sections were subsequently twice washed in 1 × TBS containing 0.025% v/v Triton-X 100, followed by 0.3% v/v hydrogen peroxide in 1 × TBS buffer for 10 min to block endogenous tissue peroxidase activity. Sections were next twice washed with 1 × TBS buffer, and a biotinylated goat anti-polyvalent secondary antibody was applied for 10 min at ambient temperature. Sections were washed four times in 1 × TBS buffer over 20 min, and streptavidin-labelled horseradish peroxidase was applied for 10 min at room temperature. Sections were subsequently rewashed four times in 1 × TBS buffer and developed for 10 min at ambient temperature using a freshly prepared 3,3′-diaminobenzidine (DAB) chromogen (30 µL added to 1.5 mL DAB substrate). Sections were washed four times for 5 min in 1 × TBS buffer before mounting with Faramount (Agilent Dako, UK). Sections were incubated away from light at 4 °C overnight before analysis.

### Haematoxylin counter-staining

Following all fluorescent labelling steps and immunostaining against phosphorylated tau coverslips were lifted from tissue sections for a final time via incubation in ultrapure water overnight. Haematoxylin Solution Gill No. 3 (Sigma Aldrich, UK) was applied directly to hydrated tissue sections (ca 200 µL) and incubated for 45 s at ambient temperature. Sections were washed in running tap water for 5 min and mounted with Faramount (Agilent Dako, UK). The mounting medium was allowed to cure fully at room temperature before analysis via brightfield microscopy.

### Microscopy

Brightfield and fluorescence microscopy was performed using an Olympus BX50 fluorescence microscope (mercury source) equipped with a BX-FLA reflected light attachment and vertical illuminator. Lumogallion fluorescence was acquired by use of a U-MNIB3 fluorescence filter cube (bandpass λ_ex_: 470–495 nm, dichromatic mirror: 505 nm, longpass λ_em_: 510 nm) and ThS fluorescence by use of a U-MWBV2 cube (bandpass λ_ex_: 440–440 nm, dichromatic mirror: 455 nm, longpass λ_em_: 475 nm, Olympus, UK). Ultraviolet (UV) induced protein autofluorescence was collected using a U-MWU2 cube (bandpass λ_ex_: 330–385 nm, dichromatic mirror: 400 nm, longpass λ_em_: 420 nm, Olympus, UK). Images were captured using an Olympus DP74 (CMOS processor) colour camera and the OLYMPUS cellSens (Standard 3.1) software suite. Optimal exposure times for collecting fluorescence micrographs were automatically set by the superfluorescence (SFL) mode of the Olympus DP74 camera. Optimised exposure settings were subsequently fixed for each corresponding autofluorescence (U-MNIB3) micrograph. Merging of fluorescence and brightfield channels was achieved using Photoshop (Adobe Systems Inc., USA).

## Results

### Biondi ring tangles and aluminium deposit in epithelial cells of the choroid plexus in a donor with late-onset epilepsy

**Figure 1:**
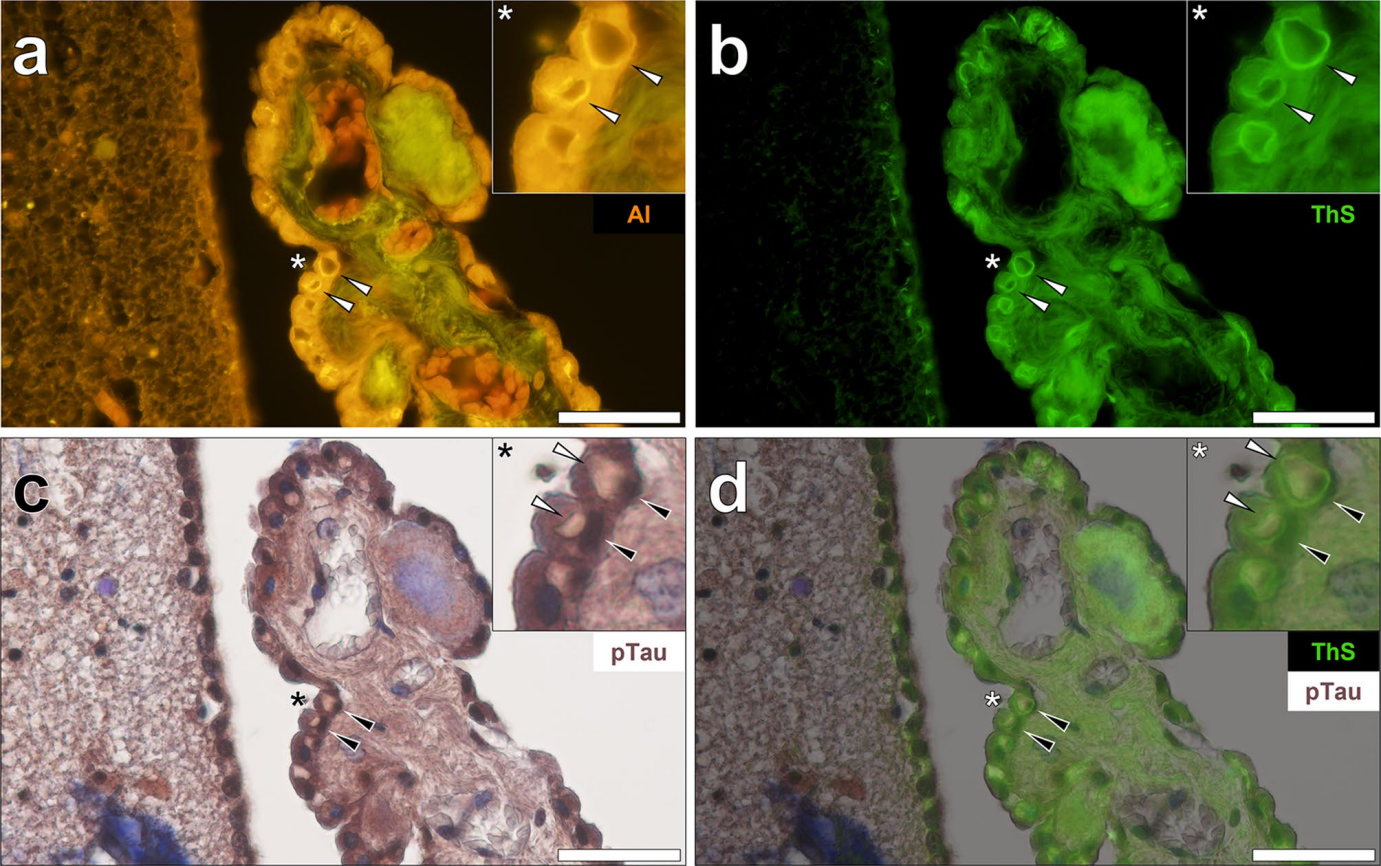
Biondi ring tangles (white arrows) in the choroid plexus adjacent to the hippocampus of a 60-year-old male donor with epilepsy. (**a**) Aluminium (Al) positive (orange) tangles in epithelial cells also produced (**b**) thioflavin S (ThS) fluorescence (green). (**c**) AT8 immunoreactive phosphorylated tau (pTau, black arrows) was deposited in the same cells lining the choroid plexus and was predominantly contained in cell nuclei. (**d**) Merging of the ThS channel revealed pTau and Biondi tangles in the same cells. Asterisks denote magnified inserts. Magnification: × 400, scale bars: 50 µm.

Lumogallion staining of the choroid plexus adjacent to the hippocampus of a male donor with late-onset epilepsy demonstrated an intense orange fluorescence emission in epithelial cells lining the choroid plexus (Fig. 1a). The intracellular fluorescence signal was notably brighter compared to cells in neighbouring cortical regions. An additional bright yellow fluorescence under the lumogallion channel revealed the presence of Biondi ring tangles. Autofluorescence of non-stained adjacent sections revealed tangle-like inclusions in the same cells that showed a weak yellow lipofuscin-like fluorescence emission (see Supplementary Fig. 1). Reactivity to thioflavin S (ThS) was noted in intracellular tangles, yielding green fluorescence adopting Biondi ring-like morphologies in the identical epithelial cells (Fig. 1b).

**Figure 2:**
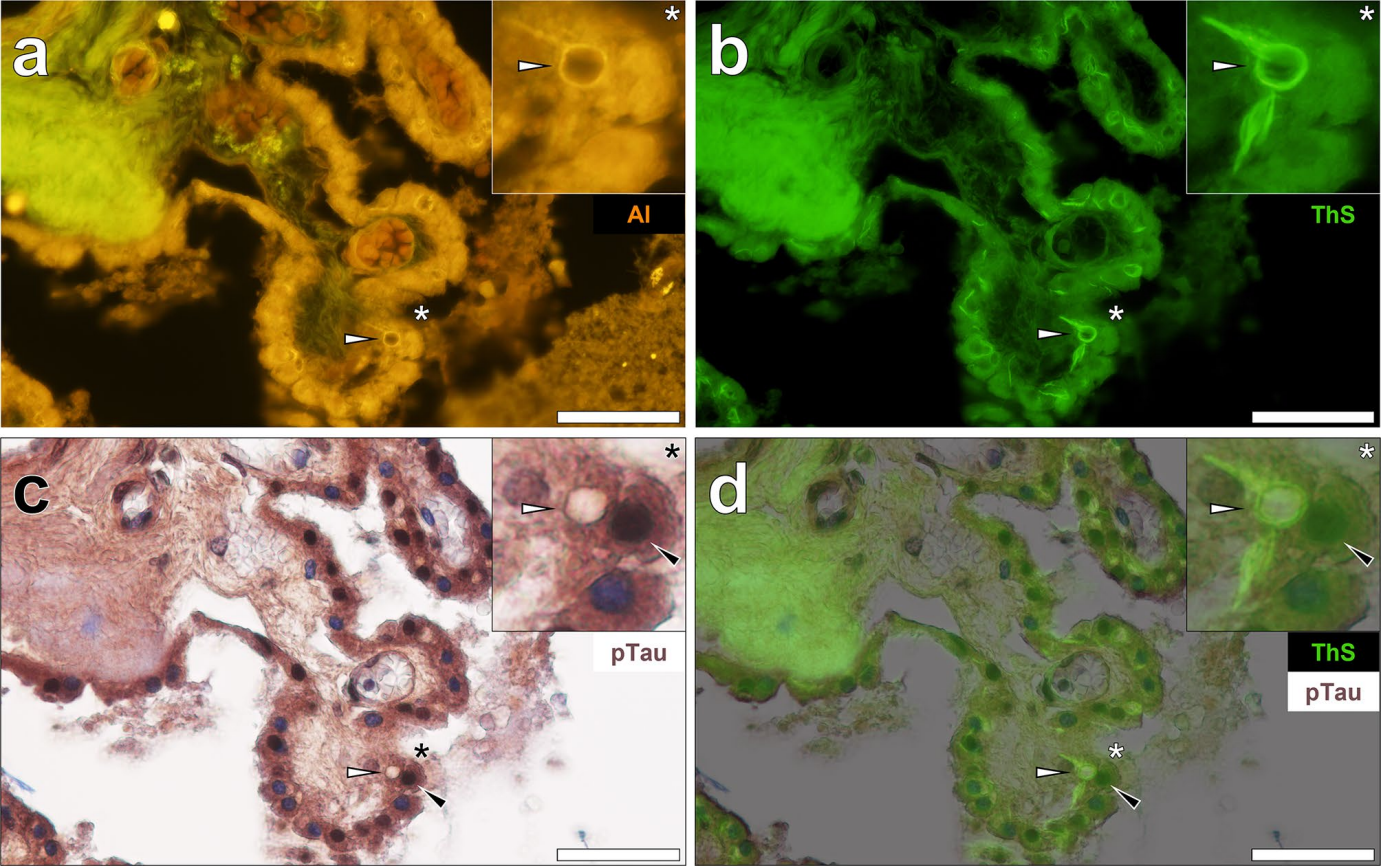
Biondi ring tangles in the cytoplasm and phosphorylated tau in cell nuclei of epithelial cells lining the choroid plexus of a 60-year-old male donor with epilepsy. (**a**) Aluminium (Al) in epithelial cells and positive fluorescence (orange) in Biondi ring tangles (white arrows). (**b**) ThS-reactivity (green) was noted in the identical tangles in cells that deposited (**c**,**d**) close to AT8-immunoreactive (black arrows) phosphorylated tau (pTau). Asterisks denote magnified inserts. Magnification: × 400, scale bars: 50 µm.

Repeated immunostainings against phosphorylated tau and counter-staining with haematoxylin consistently revealed dark brown DAB-reactive deposits in epithelial cells lining the choroid plexus (Fig. 1c). No staining was detected in the absence of the AT8 primary antibody. Haematoxylin counter-staining of AT8 immunolabelled tissue sections produced contrast enhancement of the deposited DAB chromogen (see Supplementary Fig. 2). Intense labelling for AT8-reactive tau was especially evident in cell nuclei, of which merging of the brightfield and ThS fluorescence channel showed no evident co-localisation with cytosolic Biondi ring tangles (Fig. 1d). Similar observations were noted elsewhere in the choroid plexus of the same donor where lumogallion-reactive aluminium was deposited in Biondi ring tangles (Fig. 2a) that also stained positively with ThS (Fig. 2b). Similarly, weak yellow lipofuscin-like fluorescence was observed in non-stained adjacent sections (see Supplementary Fig. 1). Dense DAB immunoprecipitation for phosphorylated tau in cell nuclei (Fig. 2c) was frequently detected in the same cells containing cytosolic Biondi ring tangles (Fig. 2d).

### Biondi ring tangles and phosphorylated tau are found in the same cells but are not co-located in the choroid plexus of two Parkinson’s disease donors

**Figure 3:**
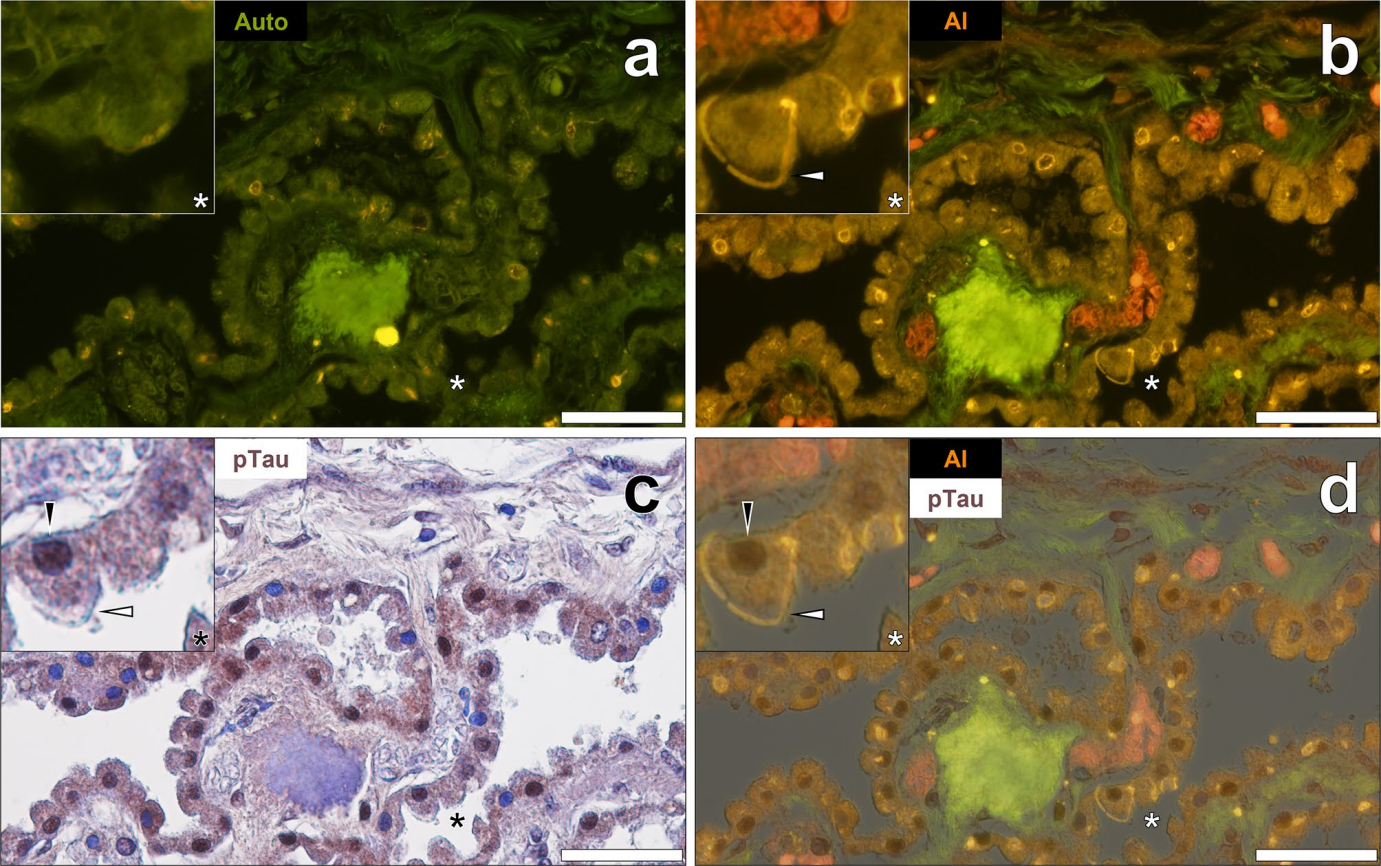
Aluminium is intricately associated with Biondi ring tangles in epithelial cells lining the choroid plexus adjacent to the hippocampus of a 67-year-old female donor with Parkinson’s disease. (**a**) Autofluorescence (green) highlighting intracellular lipofuscin-like (yellow) fluorescence. (**b**) Aluminium (Al) in cytoplasmic Biondi tangles (orange) in epithelial cells (white arrows) with (**c**) AT8 immunolabelling of phosphorylated tau (pTau) noted in cell nuclei (black arrows). (**d**) Merging of the brightfield and lumogallion fluorescence channels identified Biondi tangles and pTau in the same cells. Asterisks denote magnified inserts. Magnification: × 400, scale bars: 50 µm.

Non-stained tissue sections of the choroid plexus of a 67-year-old female with Parkinson’s disease (PD) produced a weak green autofluorescence emission when viewed under the lumogallion fluorescence channel (Fig. 3a). Similarly to hippocampal tissues taken from a donor who developed epilepsy-like seizures, yellow lipofuscin-like fluorescence identified Biondi ring-like morphologies in epithelial cells. An intense yellow/orange fluorescence emission was produced in epithelial tangles upon lumogallion staining that were seen to interdigitate between neighbouring cells (Fig. 3b). An intense and intracellular orange fluorescence emission was also noted in fenestrated capillaries in the choroid plexus stroma in PD donors. AT8 immunostaining against phosphorylated tau and counter-staining with haematoxylin demonstrated brown DAB deposition in cell nuclei (Fig. 3c) within identical cells exhibiting cytosolic Biondi ring tangles. As with a male donor who developed epilepsy, AT8-reactive tau was identified in nuclei, whereas Biondi tangles were found in the cytoplasm of the same epithelial cells lining the choroid plexus (Fig. 3d).

**Figure 4:**
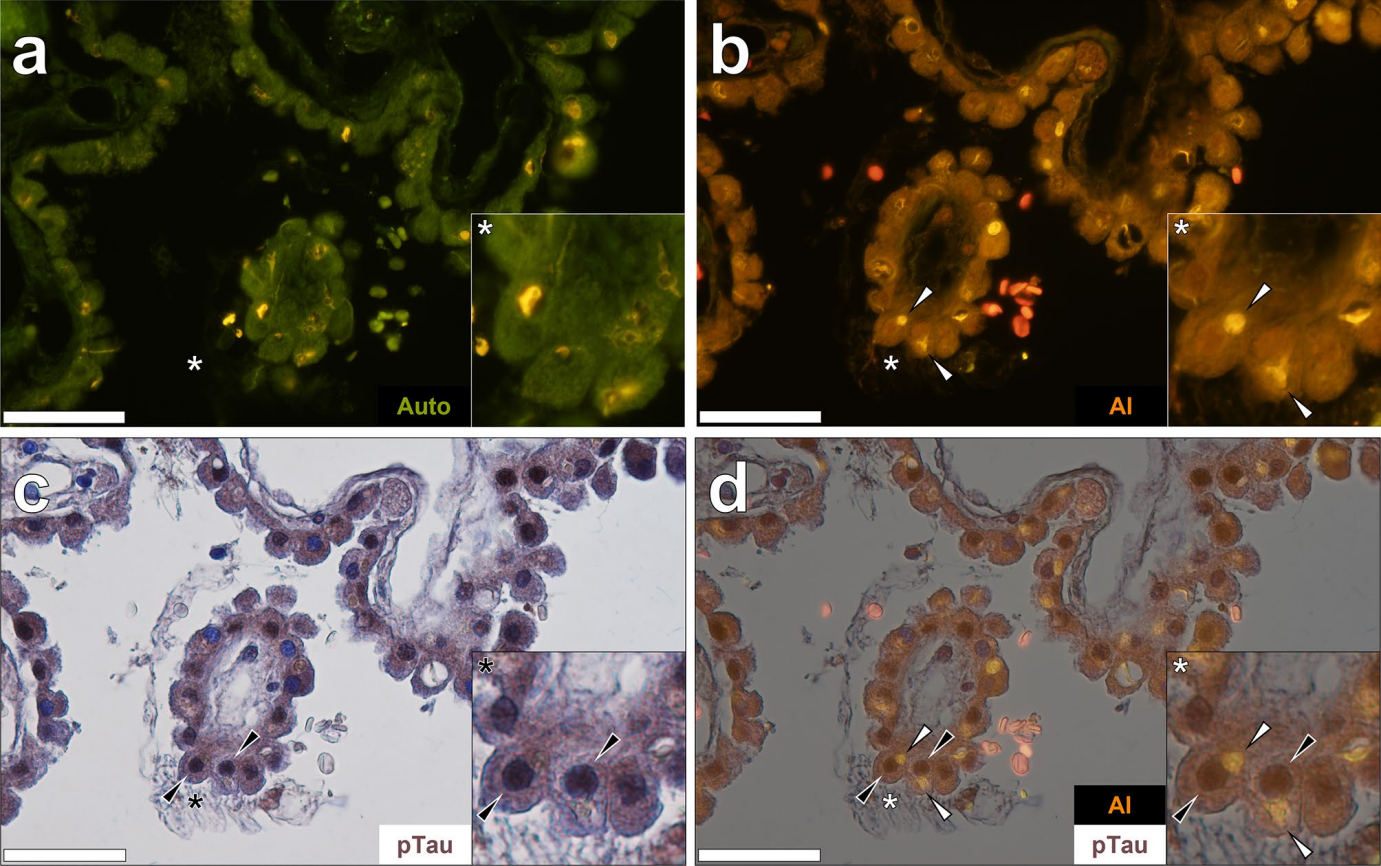
Aluminium in Biondi inclusions co-deposited with phosphorylated tau (pTau) in epithelial cells lining the choroid plexus of a 67-year-old female donor with Parkinson’s disease. (**a**) Autofluorescence highlighting intracellular lipofuscin. (**b**) Biondi inclusions (white arrows) positive for aluminium (Al, orange). (**c**) AT8 immunolabelled pTau (black arrows). (**d**) Merging of the brightfield and lumogallion fluorescence channels reveal that Biondi inclusions and aluminium are adjacently located in the same cells. Asterisks denote magnified inserts. Magnification: × 400, scale bars: 50 µm.

Lipofuscin-rich deposits were not always found to show tangle morphology. Globular Biondi-like inclusions were also observed to produce autofluorescence in the choroid plexus of the same female PD donor (Fig. 4a). Upon lumogallion staining, these globular Biondi inclusions produced an enhanced orange fluorescence emission, with Biondi ring tangles noted elsewhere in epithelial cells of the choroid plexus (Fig. 4b). DAB AT8-immunoreactivity for phosphorylated tau was noted in the nucleus of epithelial cells (Fig. 4c). AT8-reactive tau in the absence of haematoxylin counter-staining was also found to produce brown DAB labelling in the nuclear compartment of the same epithelial cells (see Supplementary Fig. 3). As with Biondi ring tangles, cytoplasmic globular Biondi inclusions were found in epithelial cells close to phosphorylated tau labelling in cell nuclei in the same female donor with PD (Fig. 4d).

### Aluminium, Biondi ring tangles and phosphorylated tau accumulate in ependymal cells in pia mater in two Parkinson’s disease donors

**Figure 5:**
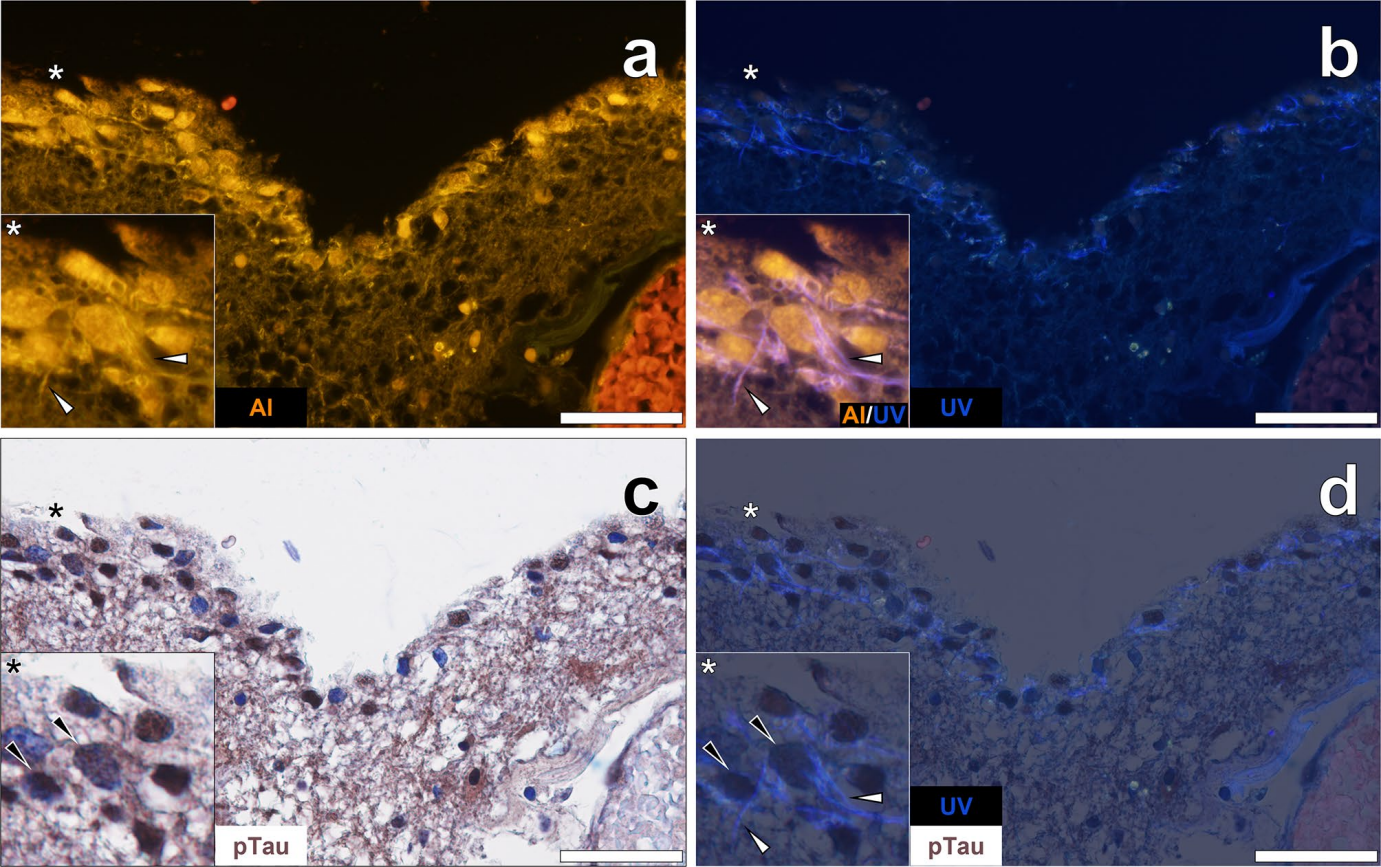
Aluminium in ependymal cells in pia mater lining the temporal horn of the lateral ventricle of a 76-year-old female donor with Parkinson’s disease. (**a**) Intracellular aluminium (Al) in ependymal cells with positive staining (orange) also noted in Biondi tangle-like cellular processes (white arrows). (**b**) Protein autofluorescence of Biondi ring tangles observed under UV illumination (merged with the lumogallion fluorescence channel in the insert). (**c**) Brown intracellular phosphorylated tau (pTau) DAB immunostaining (black arrows). (**d**) Merging of the brightfield channel with UV induced autofluorescence demonstrated proteinaceous Biondi tangles in cell cytosol with pTau localised to cell nuclei. Asterisks denote magnified inserts. Magnification: × 400, scale bars: 50 µm.

Lumogallion-reactive aluminium was frequently observed in ependymal cells in pia mater lining the temporal horn of the lateral ventricle in the choroid plexus of a 76-year-old female donor with PD (Fig. 5a). Ultraviolet (UV) illumination induced blue protein autofluorescence of identical lumogallion-stained Biondi ring tangles. Those tangles observed were found in the cytoplasm of ependymal cells and extending into the glia limitans (Fig. 5b). AT8 immunolabelling for phosphorylated tau revealed dense and often punctate deposits in ependymal cell nuclei (Fig. 5c). Ependymal cells lining the pia mater were frequently observed to produce DAB immunoreactivity to phosphorylated tau (see Supplementary Fig. 4). No apparent staining was observed in the absence of the AT8 primary antibody, and contrast enhancement of the DAB chromogen was observed upon haematoxylin counter-staining (see Supplementary Fig. 2). Merging of the brightfield and the UV autofluorescence channel demonstrated that phosphorylated tau was contained in cell nuclei of ependymal cells, with blue fluorescence of Biondi-like tangles extending into the glia limitans of adjacent pia mater (Fig. 5d).

**Figure 6:**
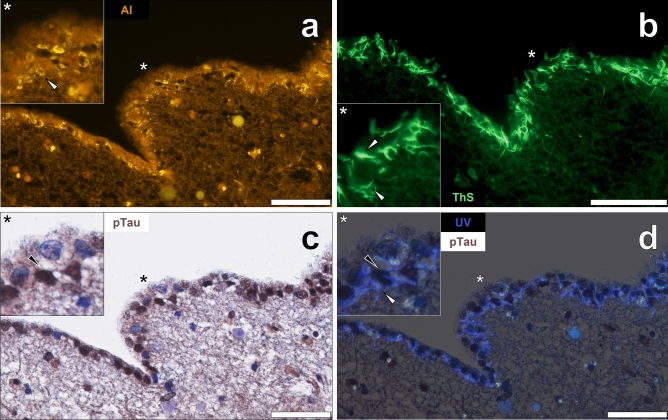
Aluminium is co-deposited with thioflavin S (ThS) reactive Biondi tangles in ependymal cells of a 76-year-old female donor with Parkinson’s disease. (**a**) Aluminium (Al) fluorescence was observed in ependymal cells of pia mater lining the lateral ventricle with intense orange/yellow fluorescence in Biondi ring tangles (white arrows). (**b**) Cytoplasmic ThS-reactive Biondi ring tangles were identified in ependymal cells in an adjacent section. (**c**) DAB immunolabelling (black arrows) of phosphorylated tau (pTau) in identical ependymal cell nuclei. (**d**) Merging of the brightfield channel with UV induced autofluorescence demonstrated proteinaceous Biondi tangles in cell cytosol with pTau localised to cell nuclei. Asterisks denote magnified inserts. Magnification: × 400, scale bars: 50 µm.

As with AT8 immunostaining for phosphorylated tau, lumogallion-reactive aluminium was frequently noted in ependymal cells in pia mater in the same female PD donor. The intracellular orange fluorescence emission in ependymal cells was of higher intensity than cells in deeper layers of the neighbouring cerebral cortex (Fig. 6a). In non-stained sections, Biondi tangles produced a weak yellow lipofuscin-like fluorescence (see Supplementary Fig. 5) and an intense orange/yellow fluorescence emission upon staining with lumogallion (Fig. 6a). ThS staining of adjacent serial sections yielded an intense green fluorescence emission highlighting Biondi ring tangles that were located in the cytoplasmic space of ependymal cells (Fig. 6b). AT8 immunolabelling of lumogallion-stained tissue sections highlighted DAB-reactive deposits of phosphorylated tau in nuclei of identical ependymal cells (Fig. 6c). In tissue sections stained solely with lumogallion, Biondi ring tangles could be readily visualised under UV illumination by a blue fluorescence emission (Fig. 6d), confirming their localisation in the cytoplasmic space of ependymal cells.

## Discussion

We have identified Biondi ring tangles in epithelial cells lining the choroid plexus and ependymal cells of the lateral ventricle in two female donors with PD and a male donor that developed late-onset epilepsy. Lipid-rich tangles were identified in both cell types via the yellow autofluorescence emission of lipofuscin in non-stained sections^26,27^. Transmission electron microscopy (TEM) of tissues excised from the choroid plexus of aged donors with sporadic AD has previously demonstrated positively stained and densely packed granules of lipofuscin interwoven with tightly packed fibrous Biondi ring tangles^23^. As a cellular hallmark of ageing, the accumulation of lipids, lipoproteins, neuromelanin and their oxidation forms punctate granular deposits of lipofuscin in cell cytosol^38,39^. Indeed, our observations of UV excitable autofluorescence in Biondi ring tangles highlighted their proteinaceous content^23^. While the presence of intracellular lipofuscin is frequently noted in neuronal cells and especially those in the brain parenchyma, these lipid-rich granules are also known to deposit in ependymal linings in ventricles^38^.

The choroid plexus is related embryologically to ependymal cells, explaining the presence of lipofuscin and Biondi-like lipofuscin vacuoles in choroidal epithelial cells^38,40^. Ultrastructural analyses of Biondi vacuoles via TEM have shown the presence of negatively stained PHFs that encapsulated lipid-droplets in choroidal epithelial cells in AD donors^41^. As lipofuscin produces fluorescence across a broad spectral range, such has necessitated the need for fluorescence quenching agents in histological analyses of human tissues^42^. However, in studies of the choroid plexus and surrounding ventricles, this phenomenon has given rise to numerous studies identifying Biondi ring tangles via their lipid-dense content^22,23,25,40,41^. Herein, lumogallion staining of the choroid plexus in two PD and one epilepsy donor identified aluminium co-localised with lipofuscin in Biondi ring tangles. Lumogallion is a sensitive and selective fluorescent molecular probe that has allowed for the unequivocal identification of aluminium in human brain tissue^36,37^. Aluminium has been observed to co-localise with cytosolic lipofuscin granules in donors with sporadic and familial AD^35,43^. Consequently, lipofuscin has been suggested to act as a ligand for aluminium, possibly explaining the high abundance of aluminium observed in Biondi ring tangles in the tissues investigated in this study^44^. Interestingly, while aluminium was readily found co-localised with lipofuscin in Biondi ring tangles in choroidal epithelial and ventricular ependymal cells, those tangles formed retained vacuole-like conformations with vacant centres.

Biondi tangles and globular inclusions have frequently been observed in the cytoplasm of choroidal epithelial cells and ventricular ependymal cells^22,23,25,41^. In hippocampal tissues from a male donor with late-onset epilepsy and in the two female PD donors, we frequently made the observation of aluminium-laden Biondi tangles and their globular inclusions in the cytoplasm of choroidal and ventricular cells. As was especially evident for the two PD donors, those tangles formed were found to interdigitate between the extracellular spaces of the tight junctions formed between epithelial cells in the choroid plexus. Similar observations have been made in aged donors without neurodegenerative or neurological disease in which SEM identified Biondi ring tangles released into the extracellular space as a possible consequence of cell rupture^22^. Herein, we made similar observations in which Biondi ring tangles were found to outwardly project from choroidal epithelia adjacent to the hippocampus of a 67-year-old female donor with PD. Lumogallion-reactive aluminium was most evident in Biondi ring tangles versus the weaker intracellular fluorescence signal observed in the choroid plexus and neighbouring ependymal cell layers. These data suggest that aluminium preferentially binds to the PHFs of Biondi ring tangles when deposited in cells of the BCSFB in the PD and late-onset epilepsy donors investigated. We have previously observed intracellular aluminium in choroidal epithelial cells of a female donor who died with a diagnosis of cerebral amyloid angiopathy (CAA), having been exposed to high levels of aluminium in their potable water supply^30^. While extensive Biondi ring tangle deposition was noted in the choroid plexus of the same male donor in this study who developed late-onset epilepsy following exposure to the same contaminated water supply, evidence for tangle association with aluminium was lacking^27^.

Aluminium is found heterogeneously distributed in human brain tissue, and its tendency to accumulate in focal or discrete deposits have made both its measurement and visualisation difficult^35,45^. While the relatively recent technique of visualising aluminium in human brain tissue has revealed a wealth of information regarding its localisation in intra- and extracellular compartments, quantitative analyses have been invaluable in measuring total aluminium brain contents in health and neurodegenerative disease. For example, we recently published a study comparing the brain aluminium content of donors with and without a diagnosis of multiple sclerosis (MS). Therein, a statistically significant and higher brain aluminium content was observed in donors with MS^46^. In further support of these findings, we found a similar association with statistically higher brain aluminium contents in donors with Alzheimer’s disease, MS and autism^47^. Herein, we have focused on the careful resection and analysis of the choroid plexus of the late-onset epilepsy and PD donors owing to the high number of Biondi ring tangles reported in the choroid plexus of donors with and without neurodegenerative disease status^11,23^.

Classical amyloid histochemistry methods, including the use of ThS and Congo red, have shown positive fluorescence and apple-green birefringence for Biondi ring tangles in the choroid plexus, respectively^40,41^. As both methods rely upon a β-pleated sheet structure for the coordination of both dye molecules, such has led to the consensus that Biondi ring tangles are comprised of misfolded proteins and hence amyloid-associated aggregates^20,23,41^. However, while early studies showed polyclonal immunoreactivity to the AD-associated Aβ peptides in Biondi ring tangles, more recent studies using monoclonal antibodies have produced minimal immunoreactivity^25^. Therein, both the PET tau tracer, ^18^F-AV-1451, and X-34, a fluorophore derived from Congo red, produced highly fluorescent labelling of Biondi ring tangles in epithelial cells of the choroid plexus of normal elderly controls and AD donors. However, minimal immunoreactivity to Aβ was found using the 4G8 monoclonal antibody that targets the epitope of residues 18–23 of the Aβ sequence^25^. The use of a pan tau polyclonal antibody confirmed that the same tangles produced positive staining for human tau. Furthermore, the monoclonal antibodies AT8 targeting phosphorylated Ser202 and Thr 205 and PHF1 targeting Ser396 and Ser404 confirmed immunolabelling against phosphorylated tau in Biondi tangles^25^.

While it has become increasingly apparent that the “off-target” labelling of ^18^F-flortaucipir is due to the coordination of the fluorophore to the PHF-like morphology of Biondi ring tangles, their principal pathogenic component is less clear^25,40^. Herein, AT8 immunolabelling identified intracellular phosphorylated tau in choroidal epithelial and ventricular ependymal cells in donors with late-onset epilepsy and PD. However, clear AT8 labelling for phosphorylated tau in Biondi ring tangles was not observed. Therefore, our results support the structure of Biondi ring tangles as unique and distinct from that of NFTs^23^. In the brains of AD donors, NFTs are 20–24 nm versus the amyloid-like 10 nm long parallel fibrils observed in senile plaques when negatively stained and analysed by TEM^23,48,49^. Therefore, the structural recognition of Biondi tangles for classical amyloid dyes, including ThS and Congo red, most likely underly their mechanistic binding to the β-pleated sheet-like fibrils of these proteinaceous inclusions.

Tau is known to redistribute from axons to the somatodendritic compartment of affected neurons in neurodegenerative diseases, including PD leading to the formation of NFTs^50^. Tau is also found in cell nuclei in neuronal and non-neuronal cells and dissociates from DNA upon its phosphorylation in the AD brain^51–53^. In donors with AD, phosphorylated tau has been identified in the stroma of the choroid plexus. The microglia associated protein, triggering receptor expressed on myeloid cells (TREM2), is highly expressed in control brains in stromal macrophages. Its decline upon ageing, especially in AD donors, contributes to impaired cellular clearance of misfolded proteins^54^. Such may explain the intracellular persistence of ubiquitinated Biondi ring tangles and NFTs of phosphorylated tau through the failures of proteasomal clearance mechanisms, common to neurodegenerative diseases including PD^1,3,41^.

Herein, we demonstrated the unequivocal association of aluminium in Biondi ring tangles in donors with PD and late-onset epilepsy. Common to both neurological conditions were intracellular tau in nuclei and Biondi ring tangles in the cytoplasm of the same cells in the choroid plexus. Diffuse cytoplasmic and AT8 positive phosphorylated tau was also evident in the identical cells. We have recently identified that aluminium is more readily associated with Aβ in extracellular senile plaques than is found co-localised to intracellular NFTs containing phosphorylated tau in the brains of donors with fAD^28,29^. Owing to the β-pleated sheet structure of Biondi ring tangles, such may explain the favourable association of aluminium for Biondi protein inclusions versus phosphorylated tau when deposited in the same epithelial and ependymal cells.

The presence of aluminium in fenestrated capillaries in the choroid plexus in PD mirrors our previous observations of aluminium crossing the meninges in donors with autism spectrum disorder^55^. Therefore, our study supports aluminium’s ability to cross the compromised BCSFB and the blood–brain barrier (BBB), respectively^56^. Aluminium is known to induce leakiness of the BBB, and in concert with Biondi ring tangles bursting into the extracellular space, such may provide a mechanism for the release of aluminium into brain parenchyma and its then likely accumulation in glial and neuronal cell types^36,37,56^. The choroid plexus is crucial to the high turnover and production of CSF^19,57^. To this end, the differentiation and maturation of multi-ciliated epithelial cells and their age-dependent expression of transporters and channels in facilitating water transport across the epithelium are crucial in this process^57^.

Chronic inflammation occurs in choroidal tissues in both PD and late-onset epilepsy, and the accumulation and persistence of intracellular aluminium would only exacerbate these deleterious effects^1,36,56,58^. Aluminium is neurotoxic, and its ability to traverse brain parenchyma by both the BCSFB and BBB will lead to its inevitable accumulation in human brain tissue^36,37,55,56^. While the small number of brain tissues analysed in this study was relatively small, relatively few studies exist regarding the presence of Biondi ring tangles in neurodegenerative disease^22,23^. Furthermore, our extensive and meticulous analysis of serial sections from each donor supports a non-phosphorylated tau component of Biondi ring tangles with the ability to act as a potential sink for aluminium. While Biondi ring tangles are considered artefacts in PET imaging studies, their ability to bind aluminium and then potentially release it upon their escape and subsequent rupture of choroidal cells may allow for a mechanism that propagates aluminium toxicity in vivo.

## Supplementary Information

Supplementary Figures.

## Data Availability

The datasets generated during and/or analysed during the current study are available from the corresponding author on reasonable request.
