# Supplementary material for: Aluminium co-localises with Biondi ring tangles in Parkinson’s disease and epilepsy
Source: Sci Rep. 2022 Jan 27;12:1465. doi: 10.1038/s41598-022-05627-8 (PMC8795119; doi:10.1038/s41598-022-05627-8)
Supplement: Supplementary file 1 — Supplementary Figures. [file 41598_2022_5627_MOESM1_ESM.pdf]

<u>SUPPLEMENTARY INFORMATION</u>

Aluminium co-localises with Biondi ring tangles in Parkinson's disease and epilepsy.

Authors: Matthew John Mold* & Christopher Exley.

Postal addresses:

The Birchall Centre, Lennard-Jones Laboratories, Keele University, Keele, Staffordshire, ST5 5BG, UK.

Running title: Aluminium in Biondi ring tangles.

Correspondence:

*Matthew John Mold PhD MRSB:	matthew.j.mold@gmail.com
Christopher Exley PhD FRSB:	drchrisexley@protonmail.com
Telephone:	+44 (0) 1782 733508

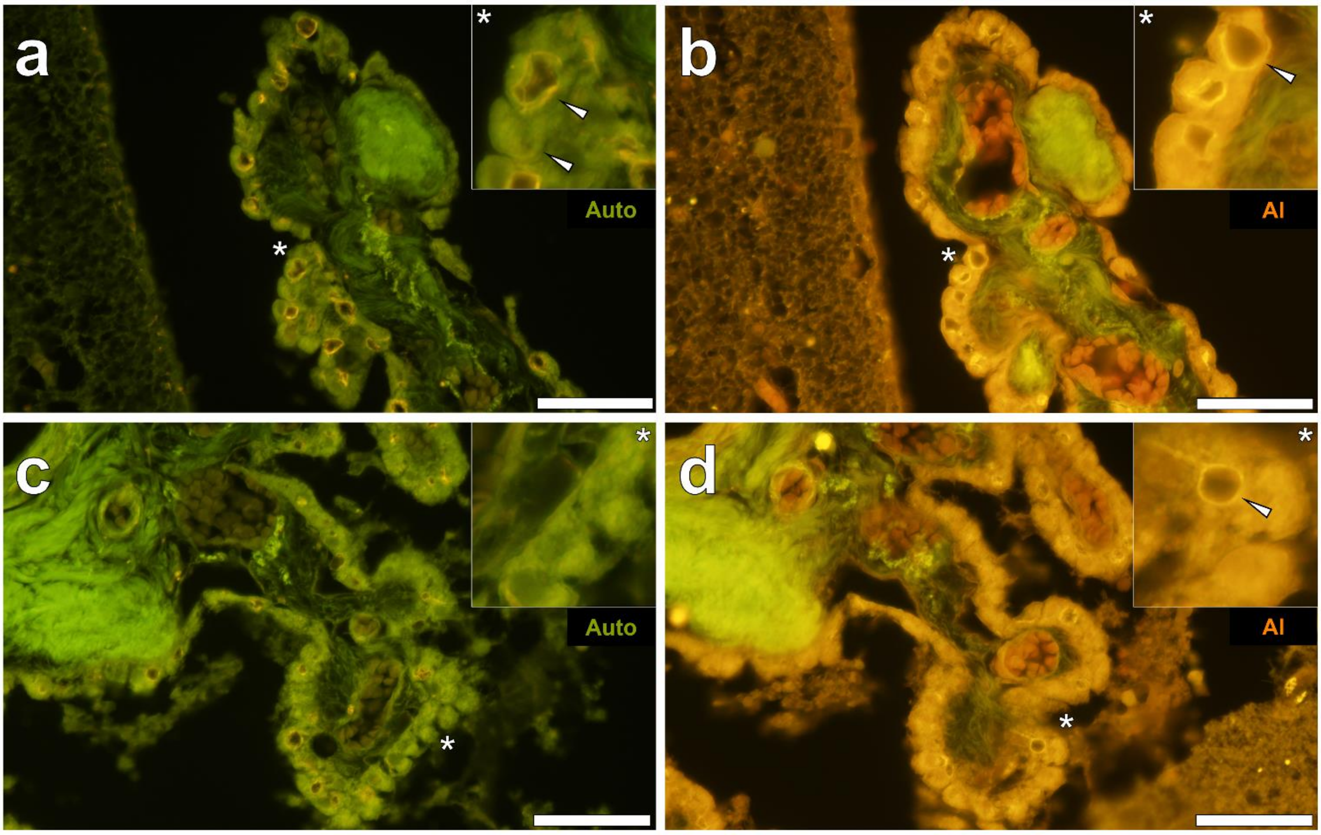

Supplementary Fig. 1. Autofluorescence and lumogallion fluorescence in adjacent sections in the choroid plexus of a donor with late-onset epilepsy (60-year-old male). (a & c) Autofluorescence demonstrating occasional intracellular lipofuscin-like fluorescence in epithelial cells resembling Biondi ring tangles (white arrows). (b & d) Lumogallion staining highlighting aluminium (Al) in Biondi ring tangles. Note the intensity of lumogallion fluorescence in epithelial cells versus neighbouring cells in the cortex. Asterisks denote magnified inserts. Magnification: X 400, scale bars: 50μm.

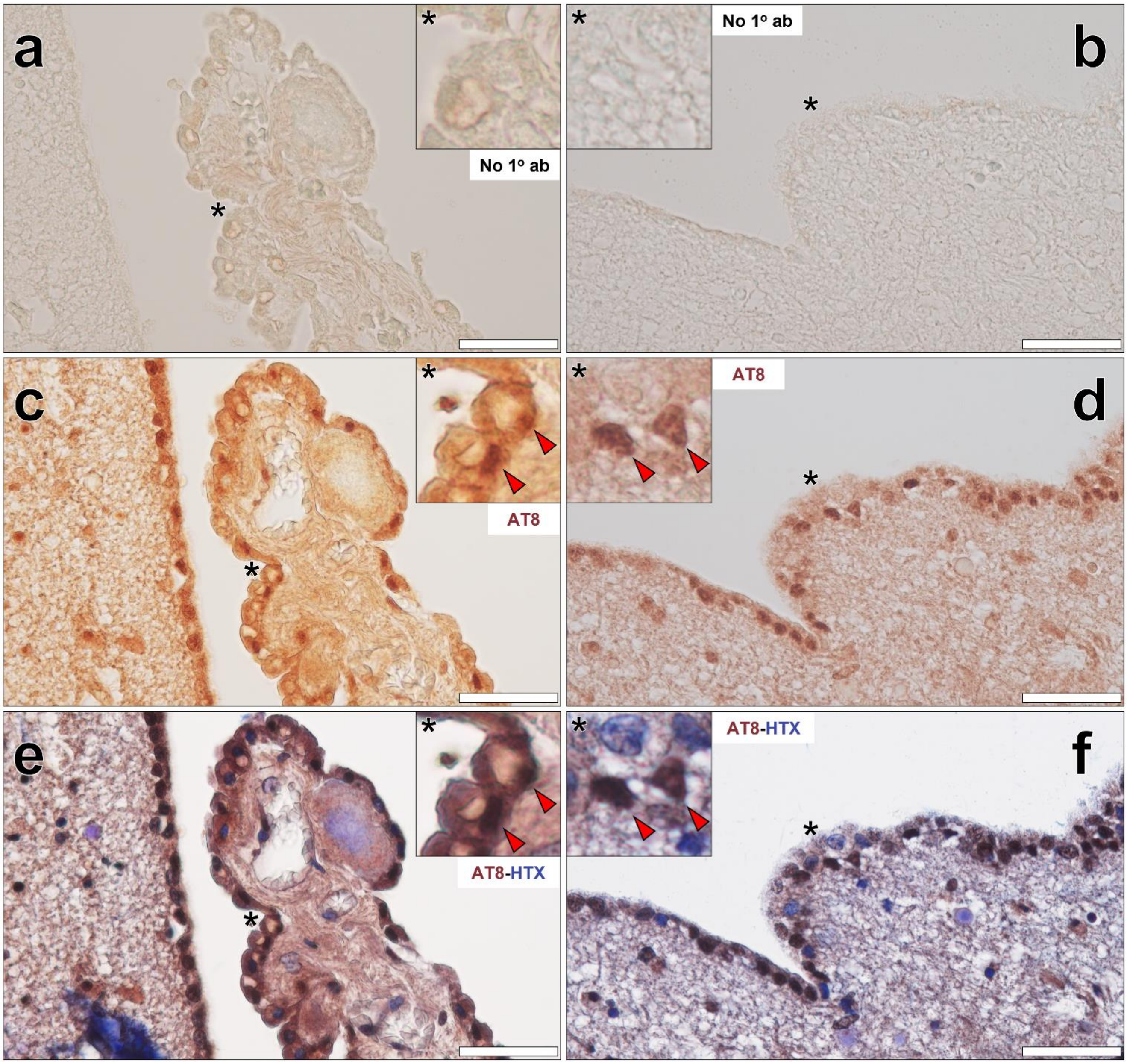

Supplementary Fig. 2. Phosphorylated tau immunolabelling observed in the absence and presence (1:50) of an AT8 primary antibody in the choroid plexus (a, c & e) of a donor with late-onset epilepsy (60-year-old male) and ependymal cells in pia mater (b, d & f) in a donor with Parkinson's disease (76-year-old female). (a & b) DAB immunostaining observed in the absence of the AT8 primary antibody. (c & d) AT8 immunostaining (brown). (e & f) Haematoxylin counter-staining (blue). Red arrows indicate positive (brown) DAB labelling of phosphorylated tau. Asterisks denote magnified inserts. Magnification: X 400, scale bars: 50µm.

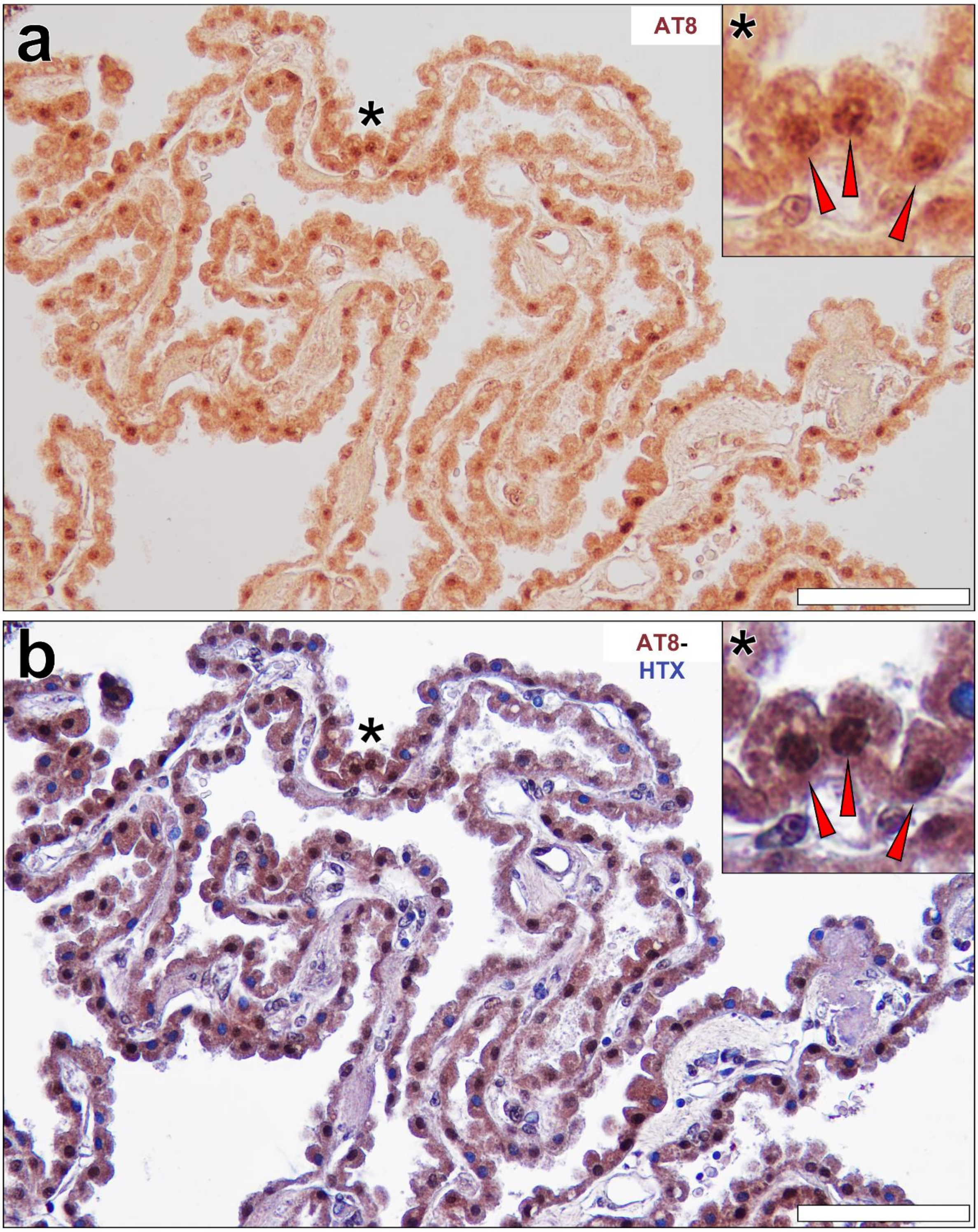

Supplementary Fig. 3. Phosphorylated tau in epithelial cells lining the choroid plexus in a Parkinson's disease donor (67-year-old female). (a) AT8 immunolabelling via secondary DAB reaction (brown) observed in epithelial cells. (b) Haematoxylin counter-staining, labelling cell nuclei (blue). In both incidents, heavy DAB immunoprecipitation was observed in cell nuclei (red arrows). Asterisks denote magnified inserts. Magnification: X 100, scale bars: 200µm.

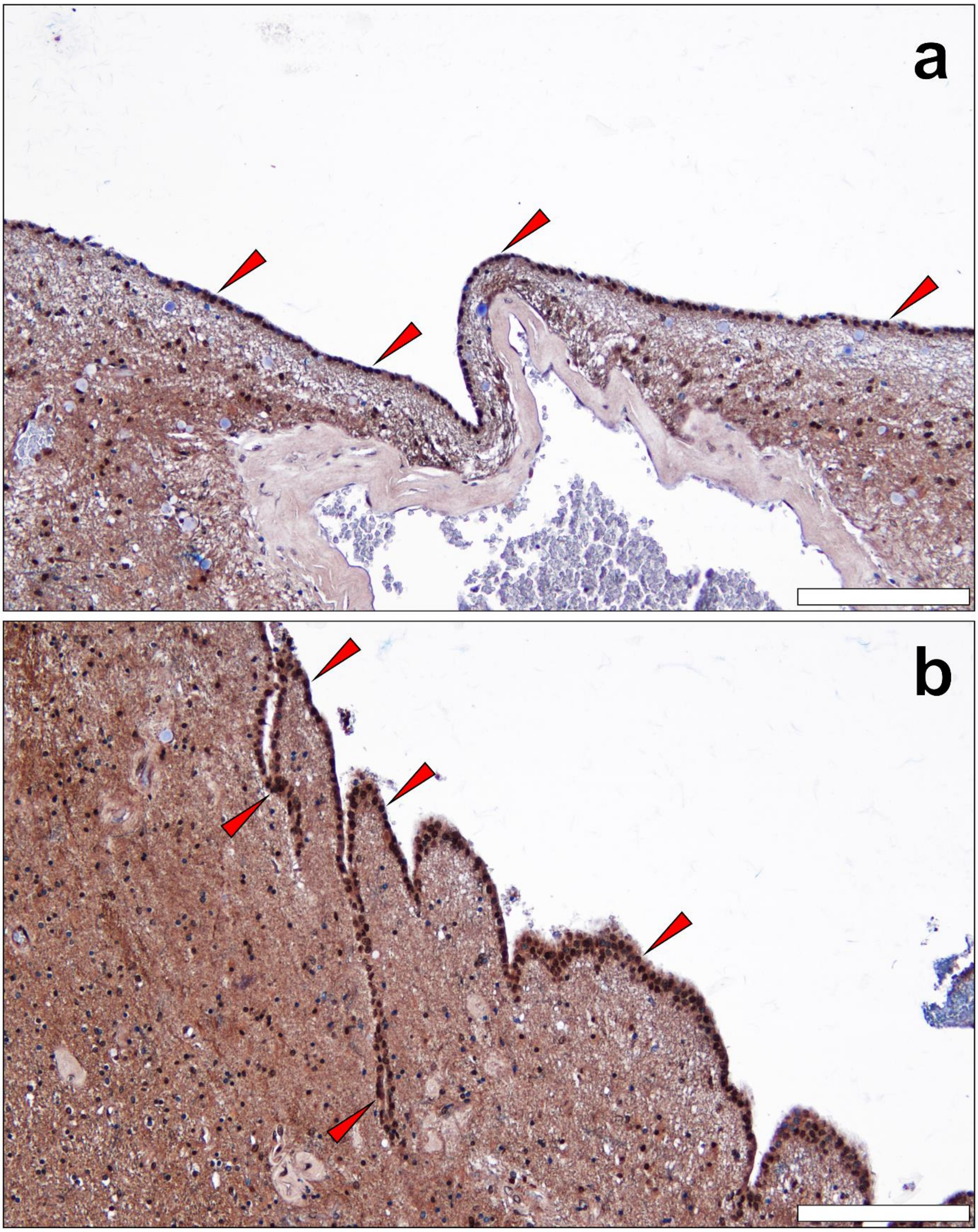

Supplementary Fig. 4. Phosphorylated tau in ependymal cells spanning pia mater of the temporal horn of the lateral ventricle in a Parkinson's disease donor (76-year-old female). **(**a & b) AT8 immunolabelling via secondary DAB reaction (dark brown) observed in ependymal cells lining the cortex adjacent to the choroid plexus (red arrows) and counter-stained with haematoxylin, labelling cell nuclei (blue). Magnification: X 100, scale bars: 200μm.

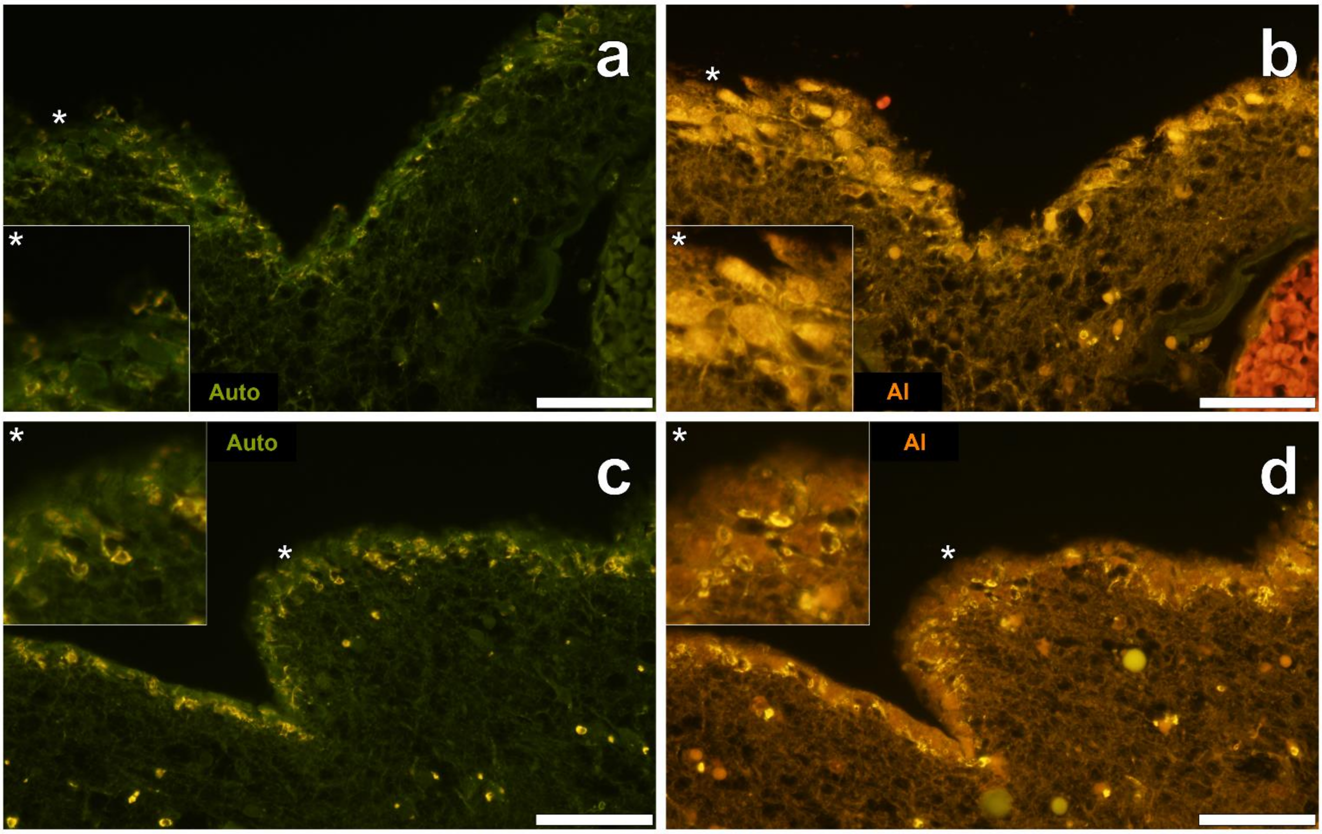

Supplementary Fig. 5. Autofluorescence and lumogallion fluorescence in adjacent sections in pia mater of the lateral ventricle in a donor with Parkinson's disease (76-year-old female). (a & c) Autofluorescence demonstrating intracellular lipofuscin-like fluorescence in ependymal cells resembling Biondi ring tangles (yellow). (b & d) Lumogallion staining highlighting intracellular aluminium (Al) in ependymal cells (orange) and cytosolic Biondi ring tangles (orange/yellow). Note the intensity of lumogallion fluorescence in ependymal cells in pia mater versus neighbouring cells in the cortex. Asterisks denote magnified inserts. Magnification: X 400, scale bars: 50µm.
